# Supplementary figures and images for: A lipoprotein partner for the Escherichia coli outer membrane protein TolC
Source: eLife. 2026 Apr 15;15:RP110666. doi: 10.7554/eLife.110666 (PMC13082787; doi:10.7554/eLife.110666)

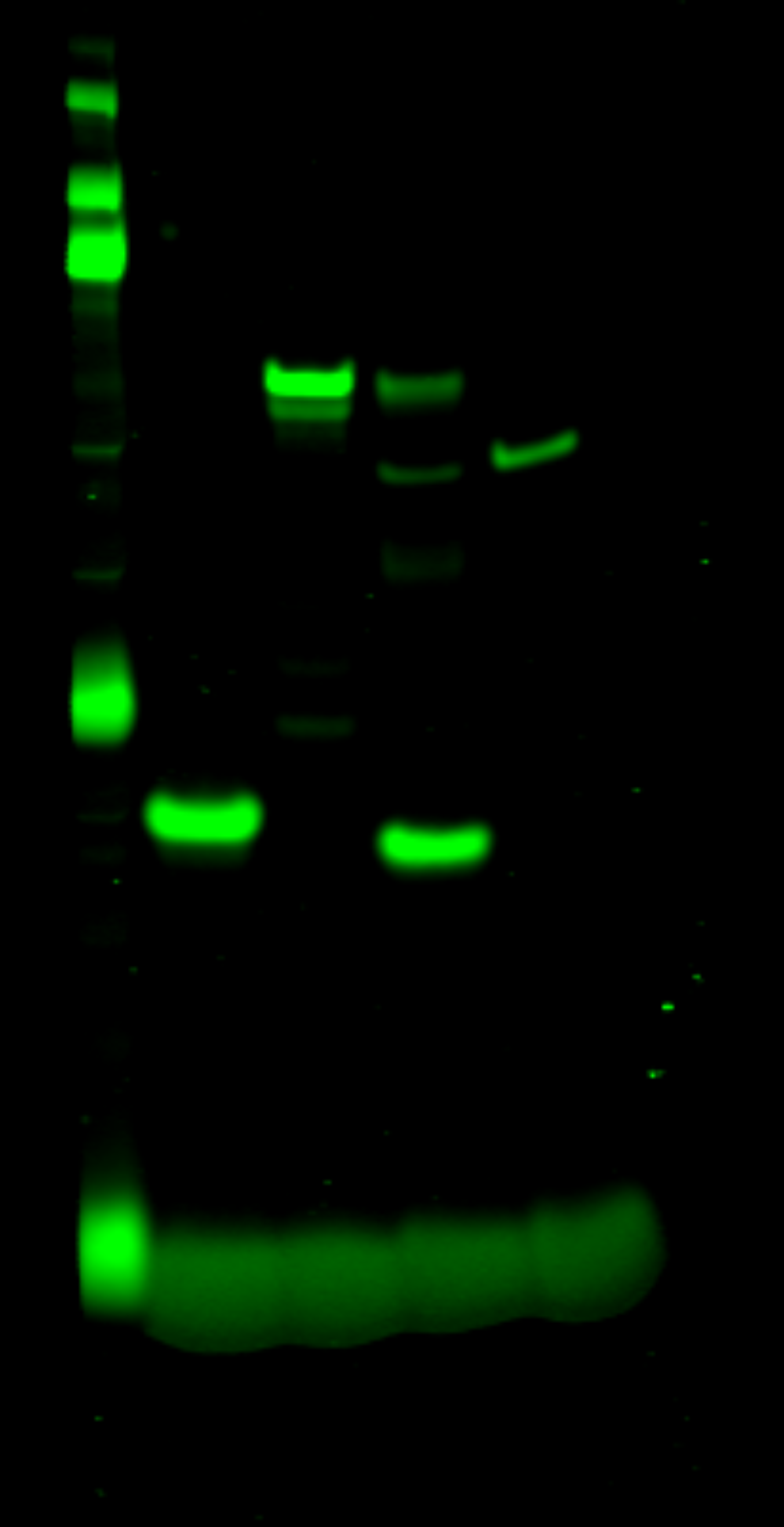

Supplement: Figure 3—source data 1. [file elife-110666-fig3-data1.zip › Folder_1_uncropped_no_label/Figure 3 supplement_source data_anti-His.tif]

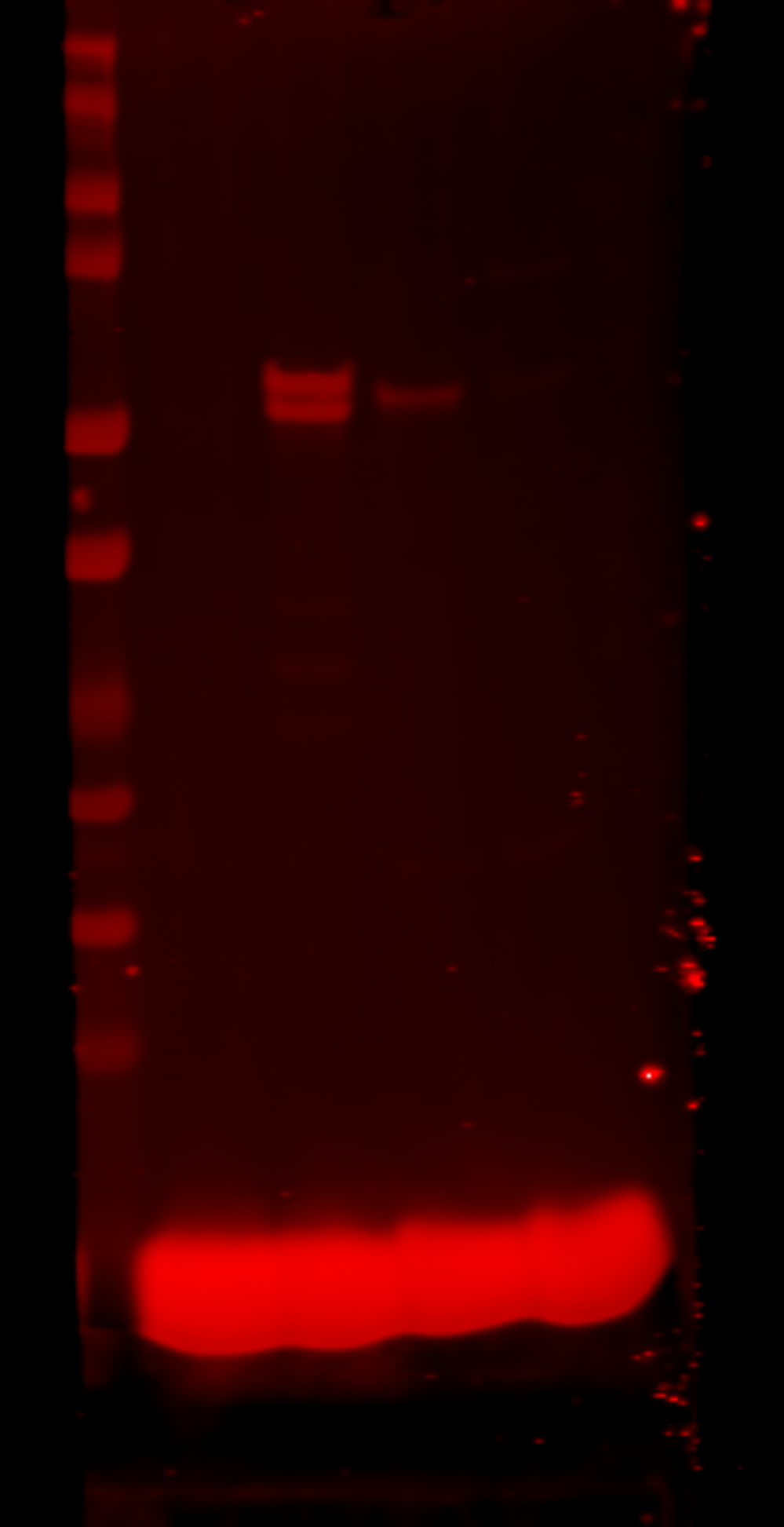

Supplement: Figure 3—source data 1. [file elife-110666-fig3-data1.zip › Folder_1_uncropped_no_label/Figure 3 supplement_source data_anti-TolC.tif]

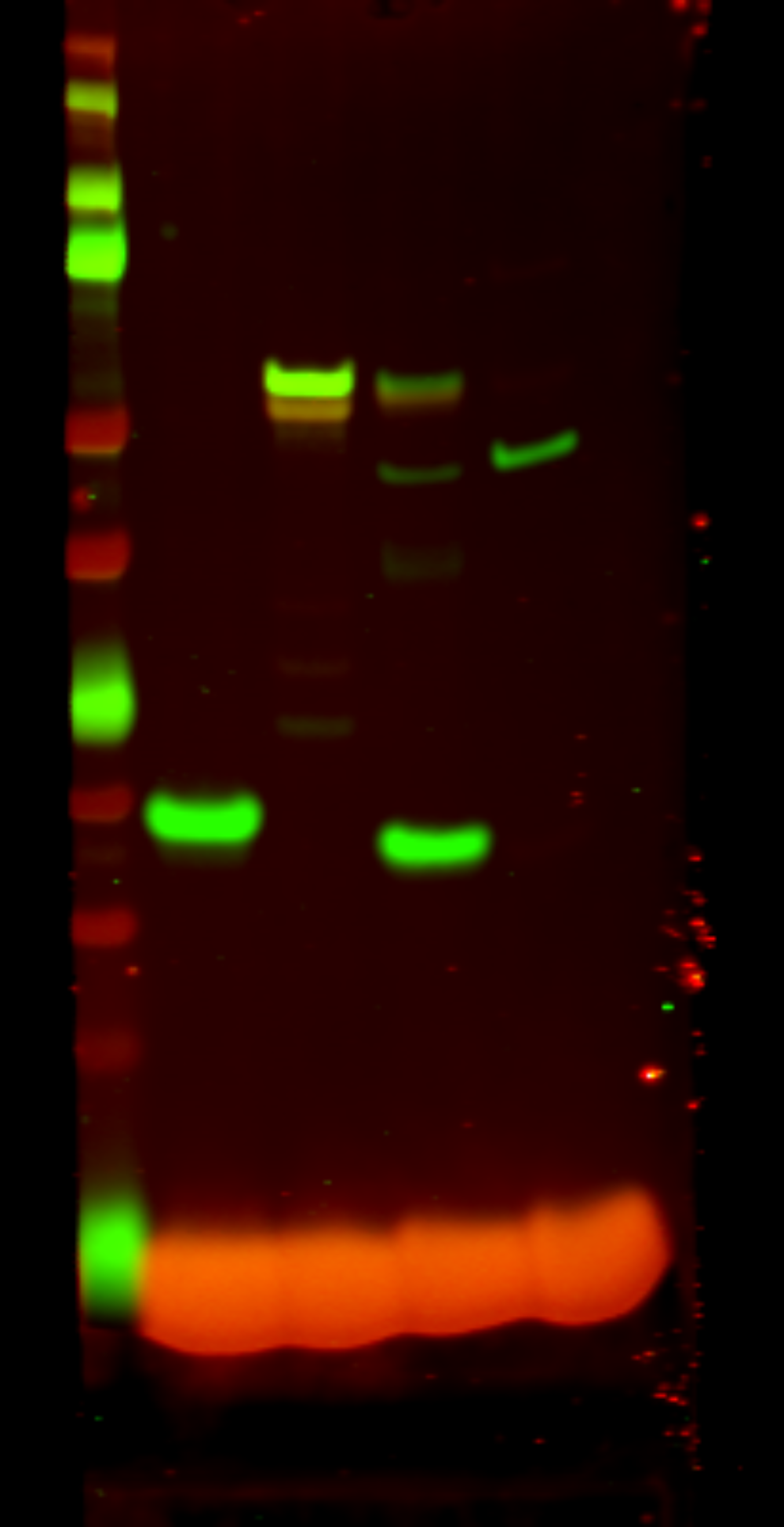

Supplement: Figure 3—source data 1. [file elife-110666-fig3-data1.zip › Folder_1_uncropped_no_label/Figure 3 supplement_source data_superposition.tif]

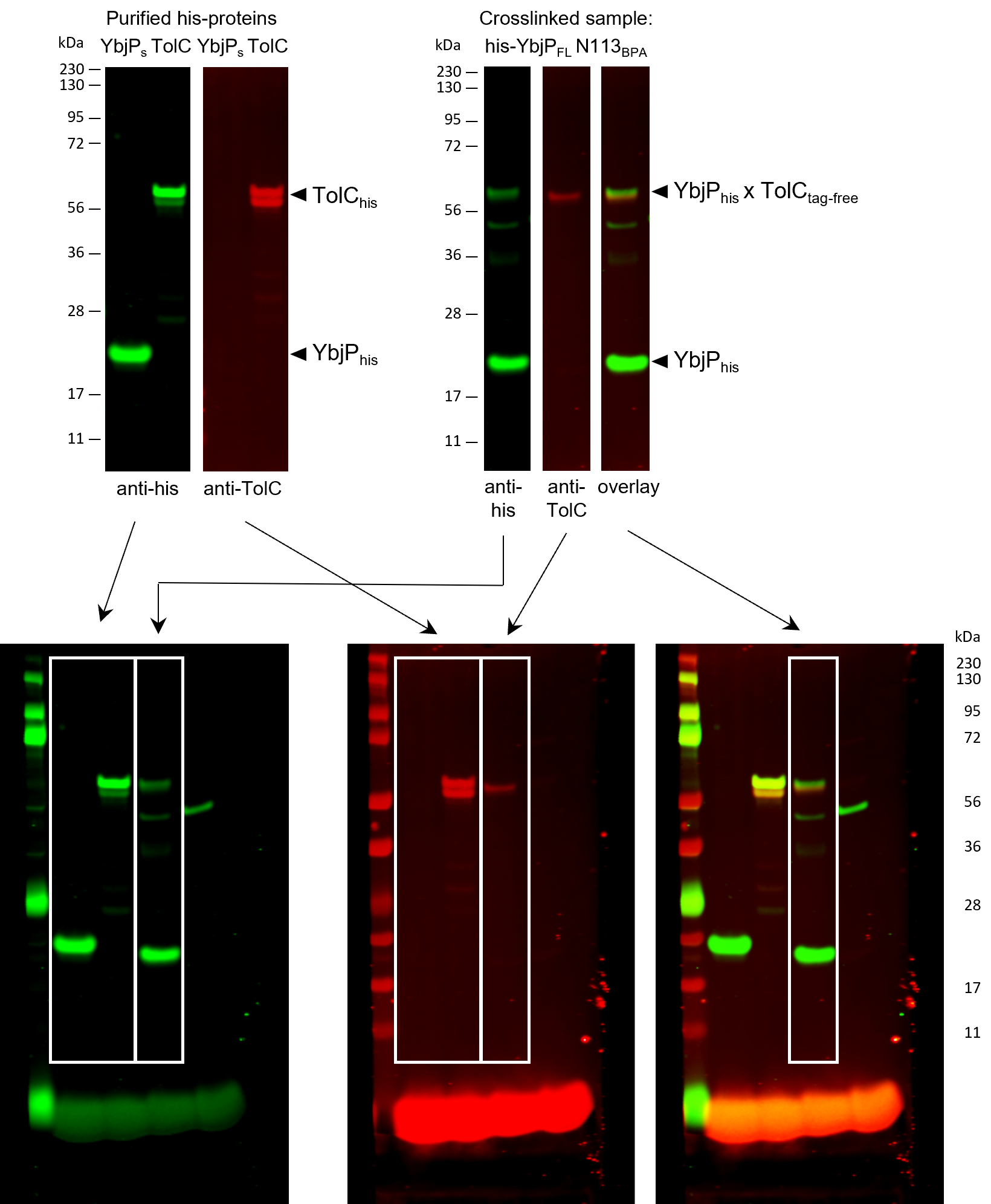

Supplement: Figure 3—source data 1. [file elife-110666-fig3-data1.zip › Folder_1_uncropped_no_label/Summary Figure 3_supplement.tif]

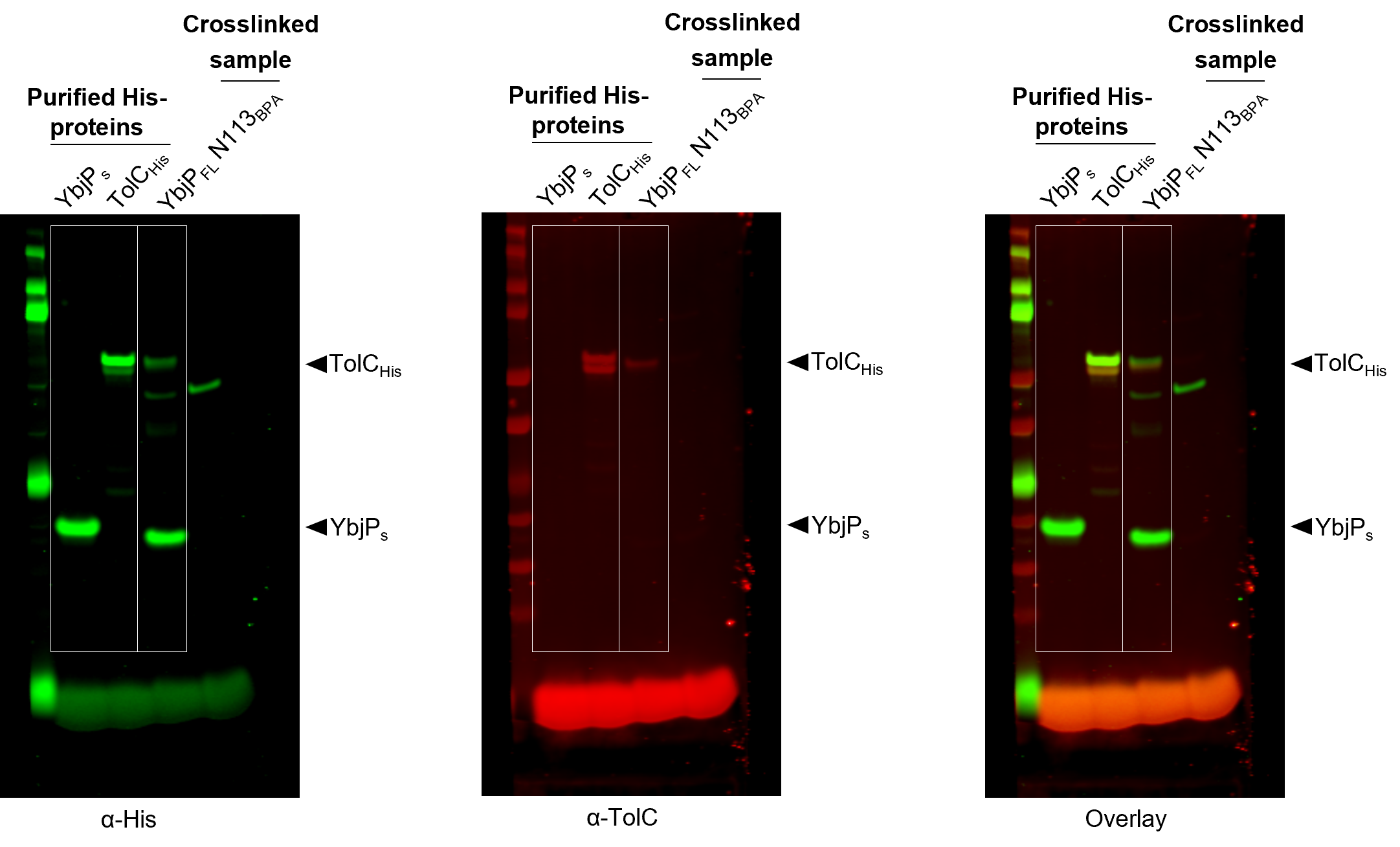

Supplement: Figure 3—source data 2. [file elife-110666-fig3-data2.zip › Folder_2_uncropped_with_labels/Figure 3 supplement_source data_with labels.tif]

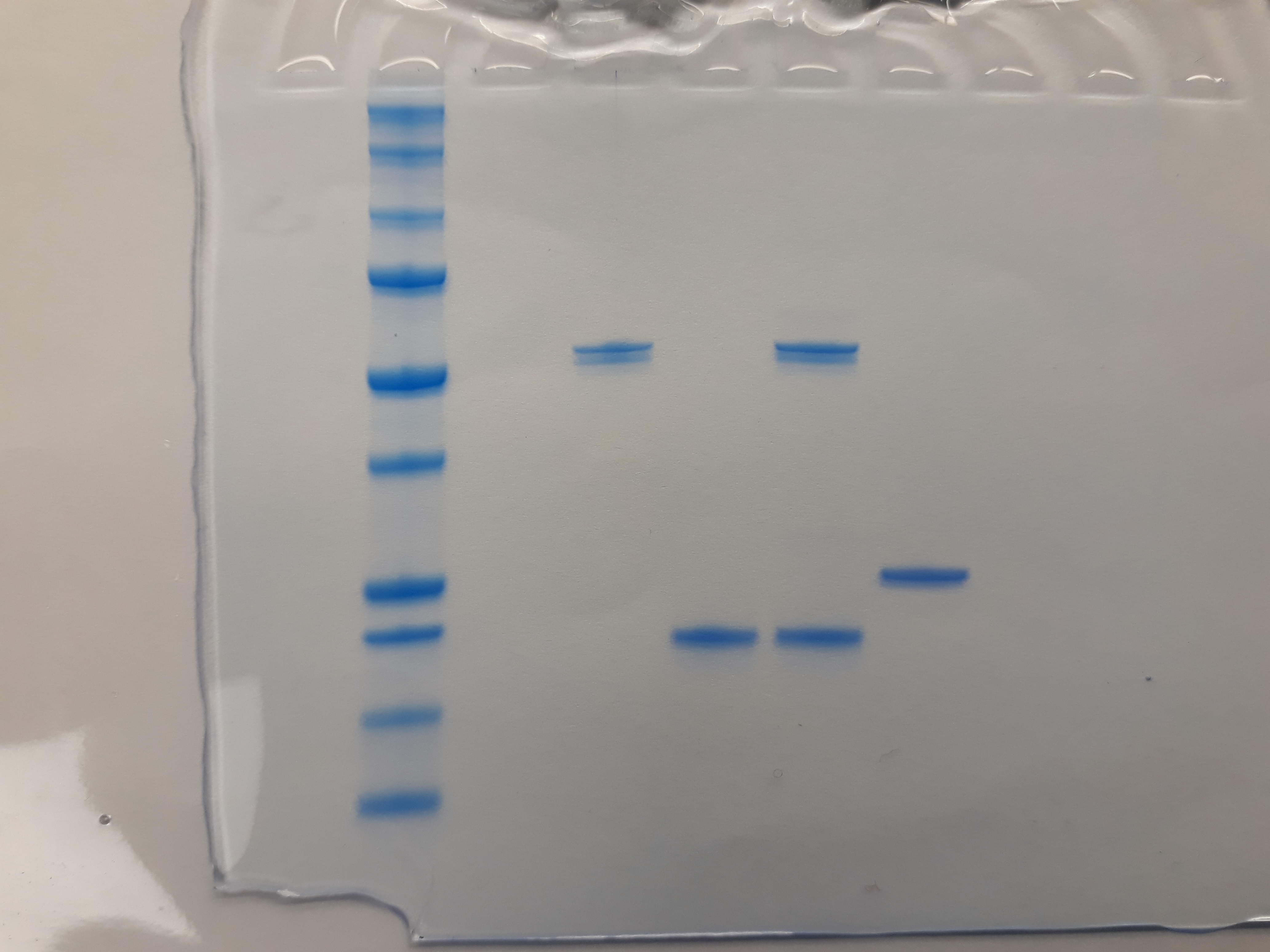

Supplement: Figure 3—figure supplement 1—source data 1. [file elife-110666-fig3-figsupp1-data1.zip › Folder_1_uncropped_no_label/Figure 3A_source data_gel_SDSPAGE_no label.jpg]

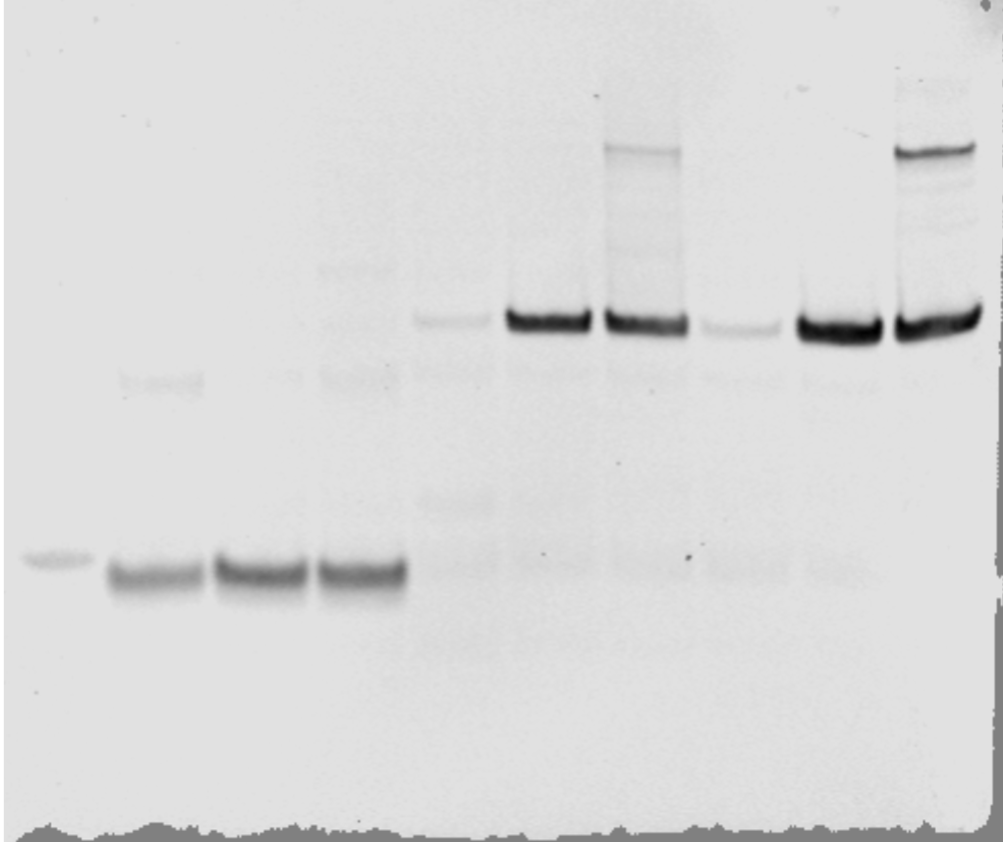

Supplement: Figure 3—figure supplement 1—source data 1. [file elife-110666-fig3-figsupp1-data1.zip › Folder_1_uncropped_no_label/Figure_3C_source data_Western_1_no label.tif]

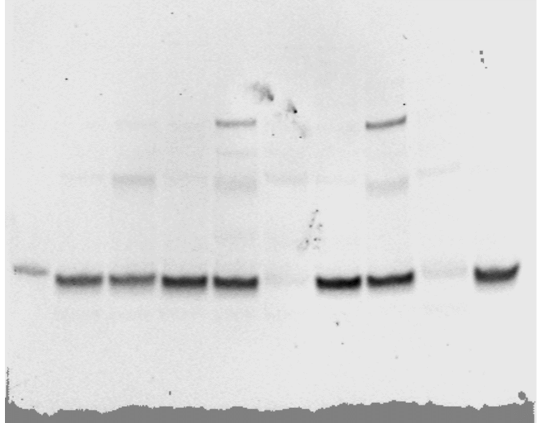

Supplement: Figure 3—figure supplement 1—source data 1. [file elife-110666-fig3-figsupp1-data1.zip › Folder_1_uncropped_no_label/Figure_3C_source data_Western_2_no label.png]

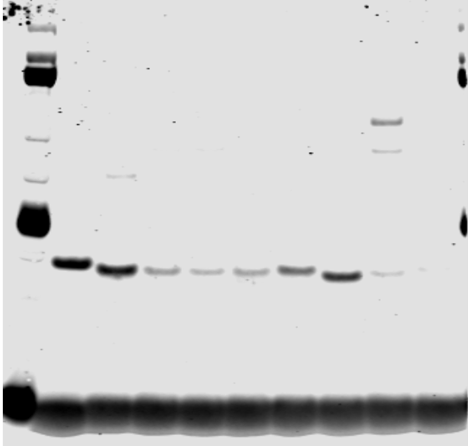

Supplement: Figure 3—figure supplement 1—source data 1. [file elife-110666-fig3-figsupp1-data1.zip › Folder_1_uncropped_no_label/Figure_3C_source data_Western_3_no label.tif]

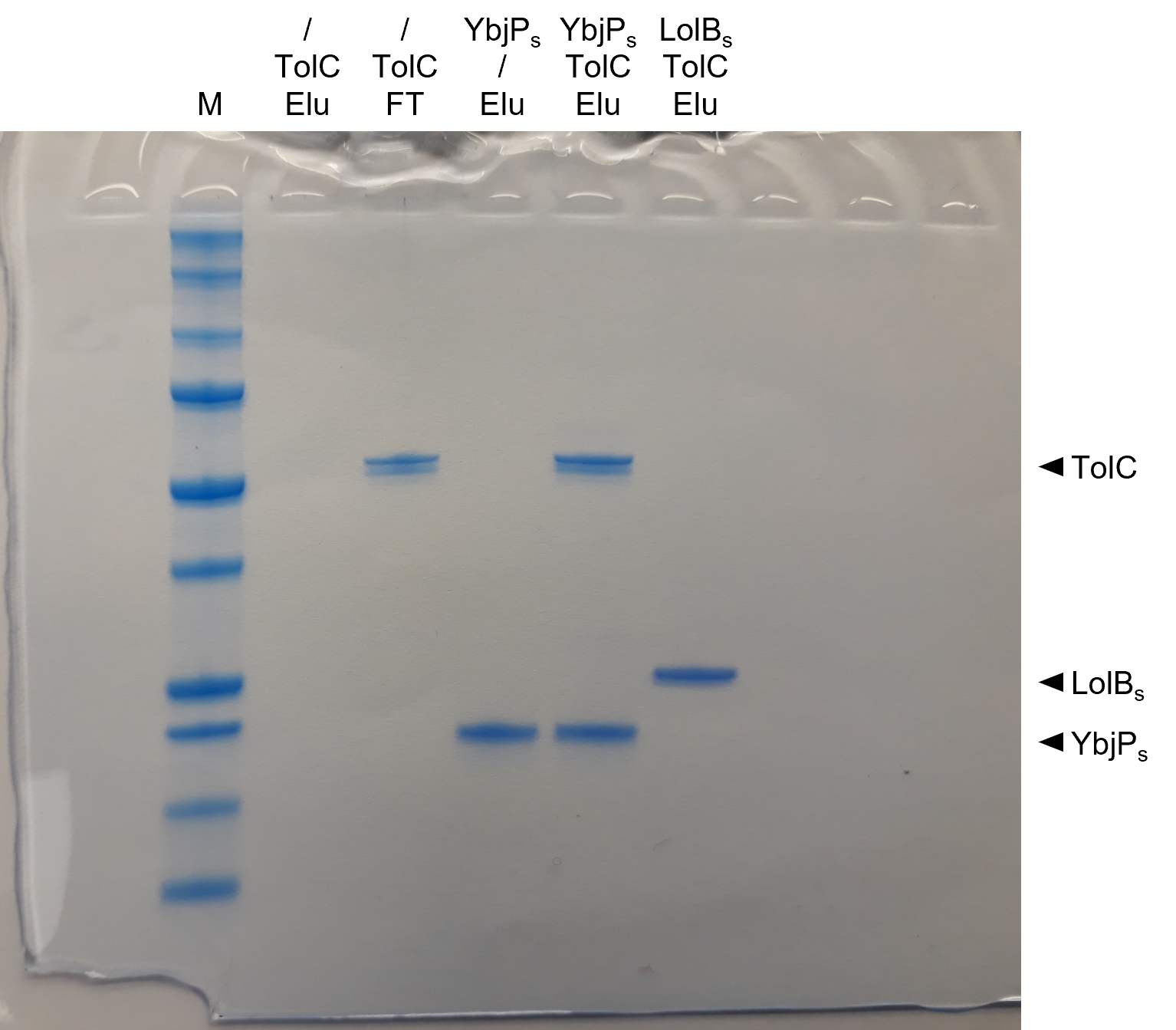

Supplement: Figure 3—figure supplement 1—source data 2. [file elife-110666-fig3-figsupp1-data2.zip › Folder_2_uncropped_with_labels/Figure 3A_source data_gel_SDSPAGE_with labels.tif]

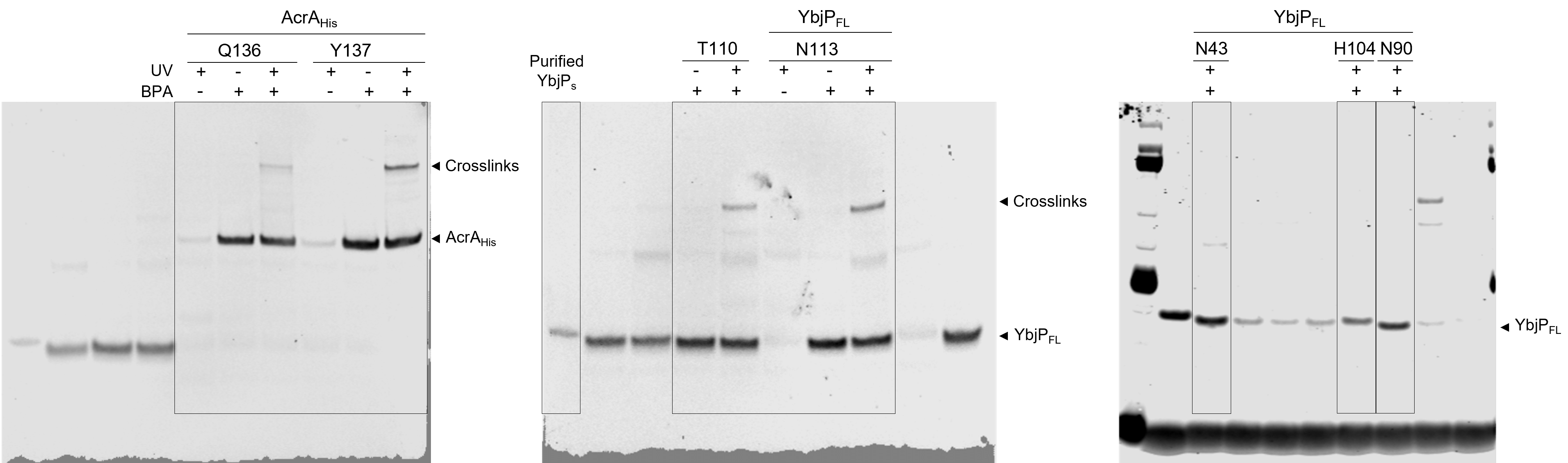

Supplement: Figure 3—figure supplement 1—source data 2. [file elife-110666-fig3-figsupp1-data2.zip › Folder_2_uncropped_with_labels/Figure 3C_source data_Western_with labels.tif]
