# Supplementary material for: A lipoprotein partner for the Escherichia coli outer membrane protein TolC
Source: eLife. 2026 Apr 15;15:RP110666. doi: 10.7554/eLife.110666 (PMC13082787; doi:10.7554/eLife.110666)
Supplement: Supplementary file 1. [file elife-110666-supp1.docx]

**Table S1. Cryo-EM data and refinement statistics for peptidisc-reconstituted MacAB-TolC-YbjP and AcrABZ-TolC-YbjP models.**

|  | **MacAB-TolC-YbjP**  **in peptidisc** | **AcrABZ-TolC-YbjP**  **in peptidisc** |
| --- | --- | --- |
| **Organism**  PDB ID | *E. coli*  9QGY | *E. coli*  9TG4 |
| EMDB ID | EMD-53150 | EMD-55890 |
| **Data collection** |  |  |
| Microscope | FEI Titan Krios | FEI Titan Krios |
| Voltage (kV) | 300 | 300 |
| Detector | K3 | K3 |
| Mode | counting, super-resolution | counting, super-resolution |
| Nominal magnification | 105,000 × | 100,000 × |
| Pixel size (Å/px) | 0.830 | 1.168 |
| Electron fluency, total (e^–^/Å^2^) | 49.75 | 56 |
| Defocus range (µm) | -1.0 to -2.4 | -1.0 to -2.5 |
| Exposure (s) | 1 | 1 |
| Frames | 40 | 40 |
| Number of micrographs | 5,399 | 13,456 |
|  |  |  |
| **Reconstruction** |  |  |
| Software | cryoSPARC-4.5.3 | cryoSPARC-4.5.3 |
| Final number of particle images | 233,775 | 97,441 |
| Point group  Map resolution, FSC_0.143_ (Å) | *C*_3_  2.48 | *C*_3_  3.17 |
| Map-sharpening B factor (Å^2^) | 76.6 | 86.3 |
| **Model composition** |  |  |
| Non-hydrogen atoms | 64,989 | 107,675 |
| Protein residues | 4,198 | 7,048 |
| Other | 3 | 3 |
| **Refinement** |  |  |
| Software | Phenix-1.20 | Phenix-1.20 |
| Correlation coefficient, masked | 0.81 | 0.82 |
| Correlation coefficient, box | 0.83 | 0.57 |
| Model resolution, FSC_0.5_ (Å)  **Validation (proteins)** | 2.7 | 3.3 |
| MolProbity score | 1.48 | 1.46 |
| Clash score | 6.73 | 6.41 |
| **Ramachandran plot statistics** |  |  |
| Favoured (%) | 97.46 | 97.45 |
| Allowed (%) | 2.54 | 2.54 |
| Outliers (%) | 0.00 | 0.01 |
