# Supplementary material for: A lipoprotein partner for the Escherichia coli outer membrane protein TolC
Source: eLife. 2026 Apr 15;15:RP110666. doi: 10.7554/eLife.110666 (PMC13082787; doi:10.7554/eLife.110666)
Supplement: Supplementary file 2. [file elife-110666-supp2.docx]

**Table S2. Thermodynamic parameters for the TolC-YbjP_s,_ interaction.** Results from isothermal titration calorimetry (ITC) were obtained with DDM-purified TolC-FLAG and N-terminally His-tagged YbjP_s_. The mean ± spread of two independent measurements is shown in bold. Experiments were carried out at 25°C. Values of ΔG, ΔH and TΔS are in kcal mol^-1^. An example of one of the thermograms and fit is shown in Figure 3B. N corresponds to the stoichiometry of the interaction.

| K_D_ (μM) | N | ΔG | ΔH | -TΔS |
| --- | --- | --- | --- | --- |
|  |  |  |  |  |
| 4.1, 5.9 | 0.64, 0.42 | -7.4, -7.1 | -37.8, -14.3 | 30.5, 7.15 |
| **5.0 ± 1.3** | **0.53 ± 0.16** | **-7.3 ± 0.2** | **-26.1 ± 16.6** | **18.9 ± 16.5** |
|  |  |  |  |  |
