# Supplementary material for: A lipoprotein partner for the Escherichia coli outer membrane protein TolC
Source: eLife. 2026 Apr 15;15:RP110666. doi: 10.7554/eLife.110666 (PMC13082787; doi:10.7554/eLife.110666)
Supplement: Supplementary file 3. [file elife-110666-supp3.docx]

**Table S3. Cryo-EM data collection statistics for AcrABZ-TolC-YbjP in peptidoglycan.**

|  | **AcrABZ-TolC-YbjP**  **peptidoglycan** |
| --- | --- |
| **Data collection and processing** |  |
| Magnification | 100,000 × |
| Voltage (kV) | 300 |
| Electron exposure (e^-^/Å^2^) | 56 |
| Defocus range (µm) | -1 to -2.5 |
| Pixel size (Å/px) | 1.168 |
| Number of micrographs | 13,456 |
| Symmetry | *C*_1_ |
| Initial particle images (no.) | 271,642 |
| Final particle images (no.) | 62,804 |
| Map resolution, FSC_0.143_ (Å) | 3.97 |
