# Supplementary material for: A lipoprotein partner for the Escherichia coli outer membrane protein TolC
Source: eLife. 2026 Apr 15;15:RP110666. doi: 10.7554/eLife.110666 (PMC13082787; doi:10.7554/eLife.110666)
Supplement: Supplementary file 4. [file elife-110666-supp4.docx]

**Table S4. Criteria for classification of DUF3828-containing proteins**

| **Family** | **Tai3** | **YbjP** | **Alphaproteobacteria** |
| --- | --- | --- | --- |
| **Taxonomy** | Betaproteobacteria Gammaproteobacteria Bacteroidota/Chlorobiota group | Enterobacterales under Gammaproteobacteria | Alphaproteobacteria |
| **Gene Synteny** | Tae3 | Arginine transporter  Amidase | Unclear |
| **Signal peptide** | Lipoprotein | Either lipoprotein or periplasmic | Periplasmic |
| **QDX motif** | Present | Absent | Present |
