# Supplementary material for: A lipoprotein partner for the Escherichia coli outer membrane protein TolC
Source: eLife. 2026 Apr 15;15:RP110666. doi: 10.7554/eLife.110666 (PMC13082787; doi:10.7554/eLife.110666)
Supplement: Supplementary file 6. — Values are presented as mean ± standard deviation from at least 5 replicates. [file elife-110666-supp6.docx]

**Table S6. Swimming motility assay**. Colony diameters of wild-type and knockout strains of *E. coli* BW25113. Values are presented as mean ± standard deviation from at least 5 replicates.

| **Strain** | **Ring diameter (mm)** | **Phenotype** |
| --- | --- | --- |
| WT | 57.2 ± 2.3 | Circular spread outwards from inoculation point |
| Δ*ybjP* | 55.4 ± 3.2 | Circular spread outwards from inoculation point |
| Δ*tolC* | 29.5 ± 8.8 | Uneven spread, biased in one direction |
| Δ*ybjP* Δ*tolC* | 29.3 ± 4.2 | Uneven spread, biased in one direction |
