# Supplementary material for: A lipoprotein partner for the Escherichia coli outer membrane protein TolC
Source: eLife. 2026 Apr 15;15:RP110666. doi: 10.7554/eLife.110666 (PMC13082787; doi:10.7554/eLife.110666)
Supplement: Supplementary file 7. [file elife-110666-supp7.docx]

**Table S7. List of primers for PCR amplification.**

| **Primer** | **Description** | **Sequence (5’ to 3’)** | **Plasmid** |
| --- | --- | --- | --- |
|  |  |  |  |
| P1 | YbjP_s__293_F | GCGCCATATGGATAACGGCACACGCAGTGGTCC | pET28-YbjP_28-171_ |
| P2 | YbjP_s__247_R | GCGCGGATCCTTAGCGATTCTCGATGGACTGACGGAGCG |  |
| P3 | YbjP_s__246_R | GCGCCTCGAGGGATTGGAAGTACAGGTTCTCGCGATTCTCGATGGACTGACGGAGCG | pET24-YbjP_28-171_ |
| P4 | YbjP_FL__250_F | GCGCCATATGCGCTACAGCAAATTGACAATGCTTATCCCC | pET24-YbjP_FL_ |
| P5 | YbjP_FL__245_R | GCGCCTCGAGGCGATTCTCGATGGACTGACGGAGCG |  |
| P6 | YbjP_FL__N43_252_F | GGAGGCCCGGATTAGGTGGCCCAGC | pET24-YbjP_FL-_N43TAG |
| P7 | YbjP_FL__N43_253_R | GCTGGGCCACGTTATCCGGGCCTCC |  |
| P8 | YbjP_FL__N90_262_F | GGAACTACTGACCTAGGATCCATTCTCC | pET24-YbjP_FL-_  N90TAG |
| P9 | YbjP_FL__N90_263_R | GGAGAATGGATCGTTGGTCAGTAGTTCC |  |
| P10 | YbjP_FL__H104_264_F | CCAGATAGCGCCTAGGTTGCCAGTGCATC | pET24-YbjP_FL-_H104TAG |
| P11 | YbjP_FL__H104_265_R | GATGCACTGGCAACGTGGGCGCTATCTGG |  |
| P12 | YbjP_FL__T110_266_F | GCCAGTGCATCGTAGATCCCTAATCGTG | pET24-YbjP_FL-_T110TAG |
| P13 | YbjP_FL__T110_267_R | CACGATTAGGGATAGTCGATGCACTGGC |  |
| P14 | YbjP_FL__N113_268_F | CGACTATCCCTTAGCGTGATGCCCG | pET24-YbjP_FL-_N113TAG |
| P15 | YbjP_FL__N113_269_R | CGGGCATCACGATTAGGGATAGTCG |  |
| P16 | AcrA_278_F | GCGCCATATGAACAAAAACAGAGGGTTTACGCC | pET24-AcrA |
| P17 | AcrA_278_R | GCGCCTCGAGAGACTTGGACTGTTCAGGCTGAGC |  |
| P18 | AcrA_Q136_280_F | CTGCTCGGTACTTAGTACATCAGTAAGC | pET24-AcrA-Q136TAG |
| P19 | AcrA_Q136_281_R | GCTTACTGATGTACTAAGTACCGAGCAG |  |
| P20 | AcrA_Y137_282_F | GCTCGGTACTCAGTAGATCAGTAAGCAAG | pET24-AcrA-Y137TAG |
| P21 | AcrA_Y137_283_R | CTTGCTTACTGATCTACTGAGTACCGAGC |  |
| P22 | LolB_s__357_F | CGCCATATGTCCGTTACCACGCCCAAAGGTCC | pET28-LolB_23-207_ |
| P23 | LolB_s__358_R | CGCGGATCCTTATTTCACTATCCAGTTATCCATTTTTA |  |
| P24 | ybjP_FRT-kanR_F | TATTATCGTAGAACGCTTTCAGAGCGATCGCTTATAAGGAAATCATTATGATTCCGGGGATCCGTCG | pJUMP29-1A-FRT-kanR |
| P25 | ybjP_FRT-kanR_R | TTTACGGGGTTTATTGGTTGATCAAGGCGTTAGCGATTCTCGATGGACTGTGTAGGCTGGAGCTGCTTC |  |
|  |  |  |  |
